# Fumigant Antifungal Activity via Reactive Oxygen Species of *Thymus vulgaris* and *Satureja hortensis* Essential Oils and Constituents against *Raffaelea quercus-mongolicae* and *Rhizoctonia solani*

**DOI:** 10.3390/biom9100561

**Published:** 2019-10-03

**Authors:** Jeong-Eun Kim, Ji-Eun Lee, Min-Jung Huh, Sung-Chan Lee, Seon-Mi Seo, Jun Hyeong Kwon, Il-Kwon Park

**Affiliations:** 1 Department of Forest Sciences, College of Agriculture and Life Sciences, Seoul National University, Seoul 08826, Korea; ionlylove7@snu.ac.kr (J.-E.K.); jie0815@snu.ac.kr (J.-E.L.); shadma@snu.ac.kr (M.-J.H.); sungchan1225@empas.com (S.-C.L.); popcon24@naver.com (S.-M.S.); sibollr.o.k@gmail.com (J.H.K.); 2 LG Household & Health Care, Seoul 07795, Korea; 3 Forest Insect Pests and Diseases Division, National Institute of Forest Science, Seoul 02455, Korea; 4 Research Institute of Agriculture and Life Science, College of Agriculture and Life Sciences, Seoul National University, Seoul 08826, Korea

**Keywords:** *Thymus vulgaris*, *Satureja hortensis*, antifungal activity, reactive oxygen species, thymol, carvacrol

## Abstract

In this study, the fumigant antifungal activity of 10 Lamiaceae plant essential oils was evaluated against two phytopathogenic fungi, *Raffaelea quercus-mongolicae,* and *Rhizoctonia solani.* Among the tested essential oils, thyme white (*Thymus vulgaris*) and summer savory (*Satureja hortensis*) essential oils exhibited the strongest fumigant antifungal activity against the phytopathogenic fungi. We analyzed the chemical composition of two active essential oils and tested the fumigant antifungal activities of the identified compounds. Among the tested compounds, thymol and carvacrol had potent fumigant antifungal activity. We observed reactive oxygen species (ROS) generation in two fungi treated with thymol and carvacrol. Confocal laser scanning microscopy images of fungi stained with propidium iodide showed that thymol and carvacrol disrupted fungal cell membranes. Our results indicated that ROS generated by thymol and carvacrol damaged the cell membrane of *R. querqus-mongolicae* and *R. solani*, causing cell death.

## 1. Introduction

Oak wilt disease caused by a fungus that is symbiotic with ambrosia beetles is a serious problem in Korea [1]. This disease was first recorded in Sungnam city, Gyeonggi Province, the Republic of Korea in 2004 and has spread to several regions of the Korean peninsula [2]. *Raffaelea quercus-mongolicae* (K.H. Kim, Y.J. Choi, & H.D. Shin) was identified as the fungal pathogen of oak wilt disease in 2009 [3]. The ambrosia beetle, *Platypus koryoensis*, transfers *R. quercus-mongolicae* to new oak trees when they attack the host tree for breeding. Although *Quercus mongolicae* (Fischer) is the main tree species attacked by *P. koryoensis*, other *Quercus* species, such as *Q. acutissima* (Carruthers), *Q. aliena* (Blume), *Q. dentate* (Thunberg), *Q. serrata* (Thunberg), and *Q. variabilis* are also affected [4]. Damping-off is a tree disease that causes serious damage in nursery gardens. Fungal pathogens in the genera *Phytopthora*, *Fusarium*, *Rhizoctonia*, and *Pythium* cause this disease [5]. Among fungal pathogens of damping-off, *Rhizoctonia solani* Kuhn occurs around the world and causes serious damage to economically important vegetables, and fruit and forest trees [6,7].

Management of the two phytopathogenic fungi mainly depends on commercial fungicides in Korea [1,2,7]. Although effective, synthetic fungicides cause harm, such as environmental contamination, toxicity to non-target organisms, and development of resistance. These issues require research to find alternatives to conventional fungicides. Several studies have investigated plant essential oils as new, safe alternatives to synthetic fungicides because the biological activities of plant essential oils and their constituents have been reported [8,9,10]. Furthermore, many plant essential oils are considered safe for humans because of their wide use in the food and fragrance market.

In this study, we investigated the fumigant antifungal activities of 10 Lamiaceae plant essential oils and their constituents against two phytopathogenic fungi*, R. quercus-mongolicae* and *R. solani*. The aim was finding new, safe alternatives for conventional fungicides. To determine the antifungal mode of action, reactive oxygen species (ROS) generation and cell membrane integrity of two phytopathogenic fungi treated with constituents derived from thyme white and summer savory plant essential oils were studied.

## 2. Materials and Methods

### 2.1. Fungal Strain and Culture Conditions

The phytopathogenic fungi *Raffaelea quercus-mongolicae* and *Rhizoctonia solani* were supplied by the National Institute of Forest Science, Seoul, the Republic of Korea. Potato dextrose agar (PDA) (Difco, IL, USA) plates were prepared using Petri dishes (diameter 90 mm), and agar-mycelial plugs (diameter 5 mm) of *R. querqus-mongalicae* and *R. solani* were inoculated into the center of the dishes. Inoculated plates were incubated at 25 °C in the dark.

### 2.2. Plant Essential Oils and Chemicals

The 10 Lamiaceae plant essential oils studied are shown in Table 1. Basil, lavender, marjoram, pennyl royal, Spanish sage, spearmint, and thyme white plant essential oils were purchased from an online retailer Jinarome (www.jinarome.com). Hyssop, patchouli, and summer savory plant essential oils were from the online retailer Oshadhi Ltd. (www.ohsadhi.eu).

Reagents (−)-α-pinene (99%), (−)-camphen (80%), (+)-camphene (80%), (−)-β-pinene (99%), (+)-β-pinene (≥98.5%), myrcene (75%), p-cymene (99%), (+)-limonene (97%), and caryophyllene oxide (90%) were from Sigma-Aldrich (Milwaukee, WI, USA). Tokyo Kasei (TCI, Tokyo, Japan) supplied (+)-α-pinene (>95%), (−)-limonene (95%), terpinen-4-ol (>95%), carvacrol (>95%), and β-caryophyllene (>90%). We obtained α-terpinene (85%), γ-terpinene (≥97%), and thymol (≥99%) from Fluka (Buchs, Switzerland), and linalool (98%) from Wako Chemicals USA (Richmond, VA, USA).

### 2.3. Fumigant Antifungal Activity Bioassays

**Figure 1:**
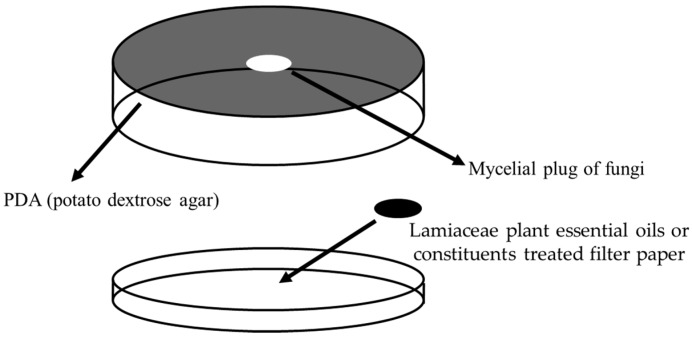
Fumigant antifungal activity test.

Fumigant antifungal activity bioassays followed the method of Kim and Park [11]. PDA (Difco, Il, USA) plates were prepared using Petri dishes (diameter 90 mm). Agar-mycelial plugs (diameter 5 mm) of *R. querqus-mongalicae* and *R. solani* were inoculated into the center of the dishes. Lamaiceae plant essential oils or their constituents in 25% dimethyl sulfoxide (DMSO, Daejung, Gyeonggi Province, the Republic of Korea) and 1% Tween 80 (Daejung, Gyeonggi Province, the Republic of Korea) were applied to a paper disc (diamter 8 mm, Advantec, Tokyo, Japan) placed on agar-free dish lids (Figure 1). Treated Petri dishes were sealed with parafilm to minimize the leaking of the test oils and compounds. The control treatment was 25% DMSO and 1% Tween 80. After treatment, plates were incubated at 25 °C in the dark. The colony diameters were measured using Vernier calipers (CD-30C, Mitutoyo Corp., Kawasaki, Japan) after mycelial growth of controls completely covered dishes (average, 2–3 days for *R. solani*, 5–7 days for *R. querqus-mongolicae*). All experiments were conducted five times. Inhibition by Lamaiceae plant essential oils or their constituents was calculated as % inhibition = C − T/C × 100 where C is a hyphal extension (mm) on control plates and T is a hyphal extension (mm) of plates treated with plant oils or constituents.

### 2.4. Gas Chromatography

Gas chromatography (GC) (7890B, Agilent, CA, USA)—with flame ionization detector (FID) and DB-5MS column (30 m × 0.25 mm i.d., 0.25 μm film thickness, Agilent, CA, USA) was used to analyze the chemical composition of summer savory and thyme white essential oils. Oven temperatures were maintained at 40 °C for 5 min, raised to 250 °C at 6 °C/min, and kept at 250 °C for 5 min. Nitrogen was the carrier gas, and the flow rate was 1.0 mL/min. The retention indices (RIs) were calculated in relation to a homologous series of *n*-alkanes (C_8_-C_22_; DB-5MS) under the same GC operating conditions [12].

### 2.5. Gas Chromatography-Mass Spectrometry

Chemical compositions of summer savory and thyme white essential oils were further determined using a gas chromatography (Agilent 7890A)-mass spectrometer (GC-MS; Agilent 5975C MSD, Agilent Technologies, Santa Clara, CA, USA) and DB-5MS column (30 m × 0.25 mm i.d., 0.25 μm film thickness, Agilent, CA, USA). The oven temperature program was the same as for gas chromatography-flame ionization detector (GC-FID) analysis. Helium was the carrier gas, and the flow rate was 1.0 mL/min. The effluence from the DB-5 MS column was introduced directly into mass spectrometer (MS) through a transfer line (280 °C). The ionization was by electron impact (70 eV, source temperature 230 °C). The scan range was 41–400 amu. Most compounds of summer savory and thyme white plant essential oils were tentatively identified by comparing the mass spectra of the peaks with authentic samples in the National Institute of Standards and Technology mass spectral (NIST MS) library.

### 2.6. Assessment of Reactive Oxygen Species Generation

Intracellular ROS generation of *R. quercus-mongolicae* and *R. solani* was observed using the ROS-sensitive dye 2′,7′-dichlorofluorescin diacetate (≥97, Sigma-Aldrich, MO, USA). Nutrient agar (NA) (Difco, IL, USA) plates were prepared in Petri dishes (diameter 90 mm). Agar-mycelial plugs (diameter 5 mm) of *R. querqus-mongalicae* or *R. solani* were placed in the center of the dishes for incubation at 25 °C (one day for *R. solani* and four days for *R. querqus-mongalicae*). On agar-free lids, paper discs (diameter 8 mm, Advantec, Tokyo, Japan) with 10 mg carvacrol and thymol were placed. Controls received only 25% DMSO and 1% Tween 80. After treatment, treated and control NA plates were incubated for one day at 25 °C. After incubation, plugs from treated and control NA plate were stained with 3 μM 2′,7′-dichlorofluorescein diacetate for 1 h at 37 °C in the dark. Stained plugs were washed with phosphate-buffered saline (PBS, Sigma-Aldrich, MO, USA) diluted 10 times with distilled water, and observed with a confocal laser scanning microscope (CLSM) (excitation: 488 nm, emission: 490-560 nm, SP8X, Leica Microsystems, Germany)..

### 2.7. Cell Membrane Integrity Assay

Cell membrane integrity of *R. quercus-mongolicae* and *R. solani* was measured by using propidium iodide (PI) (Thermofisher, MA, USA). PDA plates were prepared using Petri dishes (diameter 90 mm). Agar-mycelial plugs (diameter 5 mm) of *R. querqus-mongalicae* and *R. solani* were placed in the center of dishes for incubation at 25 °C (one day for *R. solani* and four days for *R. querqus-mongalicae*). On agar-free lids, paper discs with 10 mg carvacrol and thymol were placed. The controls only received 25% DMSO and 1% Tween 80. After treatment, treated and control PDA plates were incubated for one day at 25 °C. After incubation, plugs from PDA plates were stained with PBS 1 mL and PI (0.00625 μL for *R. querqus-mongalicae* and 0.05 μL for *R. solani*) for 15 min at 25 °C. Stained plugs were observed under CLSM (excitation: 561 nm, emission: 570–650 nm, SP8 X, Leica Microsystems).

### 2.8. Statistical Analysis

Antifungal activities of plant essential oils or their constituents were determined and transformed to arcsine square root values before analysis of variance. Treatment means were compared and separated by Tukey’s HSD test. Means ± standard error (SE) values for untransformed data were reported. We used R (ver. 3.5.1. R foundation for statistical computing, Austria) with R studio (ver. 1.1.456, R Studio Inc. MA, USA) for statistical analysis. Tukey’s HSD tests were performed with R package agricolae [13].

## 3. Results and Discussion

### 3.1. Fumigant Antifungal Activities of Lamiaceae Plant Essential Oils

Fumigant antifungal activities of 10 Lamiaceae plant essential oils against *R. querqus-mongolicae* are in Table 2. Among test oils, summer savory and thyme white essential oils showed the most potent antifungal activity. The activity was 91.50% for summer savory and 73.52% for thyme white oil at 1.25 mg/paper disc concentration and 54.40 and 50.18% at 0.625 mg/paper disc concentration, respectively. Spearmint plant essential oil had strong fumigant antifungal activity at 10 mg/paper disc concentration and moderate or weak fumigant antifungal activity at ≤5 mg/paper disc concentration. Antifungal activity of pennyroyal was 72.38% at 10 mg/paper disc concentration and 44.40% at 5 mg/paper disc concentration. Other plant essential oils had moderate or weak fumigant antifungal activity against *R. querqus-mongolicae* at 10 mg/paper disc concentration.

Fumigant antifungal activities of 10 Lamiaceae plant essential oils against *R. solani* are in Table 3. Among the tested oils, summer savory and thyme white essential oils showed 100% fumigant antifungal activity against *R. solani* at least 2.5 mg/paper disc concentration. The fumigant the antifungal activity of summer savory was higher than that of thyme white at 0.625 mg/paper disc concentration. However, antifungal activity of thyme white essential oil was stronger than that of summer savory at 0.3125 mg/paper disc concentration. Antifungal activities were 41.98% for summer savory and 54.38% for thyme white at 0.3125 mg/paper disc concentration. Antifungal activities were 87.96% for pennyroyal and 91.30% for spearmint at 5 mg/paper disc concentration and 36.42% and 44.18% at 2.5 mg/paper disc concentration, respectively. Other plant essential oils had moderate or weak fumigant antifungal activity against *R. solani* at 5 mg/paper disc concentration. The Lamiaceae family is distributed widely around the world and has great economic value [14]. Fungicidal activity of thyme white essential oil against two phytopathogenic fungi, *Phytophthora cactorum,* and *Cryponectria parasitica* has been reported [15]. Abdollahi et al. [16] insisted that thyme and summer savory plant essential oils exhibited strong antifungal activity against two postharvest fungi, *Penicillium digitatum,* and *Rhizopus stolonifera*. Although many antimicrobial activities of plant essential oils belonging to the Lamiaceae family have been reported [15,16,17], no studies report on the fumigant antifungal activity against *R. querqus-mongolicae* and *R. solani*.

### 3.2. Chemical Composition of Summer Savory and Thyme White Essential Oils

The chemical compositions of the active essential oils of summer savory and thyme white were analyzed using GC and GC-MS (Table 4). The most abundant compound in summer savory oil was carvacrol (39.84%) followed by *γ*-terpinene (34.63%), *p*-cymene (10.72%), *α*-terpinene (3.51%), and *β*-caryophyllene (2.40%). Other compounds were less than 2% of the oil. In thyme white essential oil, *p*-cymene (30.16%) was the most abundant compound followed by thymol (25.32%), *γ*-terpinene (11.44%), linalool (9.49%), α-pinene (5.25%), and carvacrol (3.46%). Other compounds, such as *β*-caryophyllene, camphene, and limonene were less than 2% each. The chemical compositions of summer savory and thyme white have been reported [18,19]. Moosavi-Nasab et al. [18] reported that the main components of *S. hortensis* oil were carvacrol at 54.7% and *γ*-terpinene at 26.9%. These results were consistent with our study. Zambonelli et al. [19] reported the chemical composition of *Thymus vulgaris* plant oils extracted from whole plants cultivated in different geographical areas of Italy and France. Thymol and *p*-cymene were two main components of thyme oil, although the most abundant component differed according to the area. The composition of carvacrol was low in all analyzed thyme oils. Although a few differences were observed in the chemical composition of the components in summer savory and thyme white essential oils between our study and prior studies [18,19], the major components were the same. The dates of harvest, storage period, extraction method, or climate could influence the chemical compositions of plant essential oils [19,20].

### 3.3. Fumigant Antifungal Activities of Constituents from Summer Savory and Thyme White Essential Oils

Fumigant antifungal activities of constituents from summer savory and thyme white plant essential oils against *R. quercus-mongolicae* and *R. solani* are in Table 5; Table 6. Among test compounds, thymol and carvacrol exhibited the strongest fumigant antifungal activities against the phytopathogenic fungi. In a test with *R. querqus-mongolicae*, fumigant antifungal activities were 100% for thymol and 81.28% for carvacrol at 0.625 mg/paper disc and 53.30% and 30.68% at 0.3125 mg/paper disc, respectively. Terpinen-4-ol had moderate antifungal activity at a concentration of more than 0.625 mg/paper disc, but weak antifungal activity at 0.3125 and no activity at 0.15625 mg/paper disc. Other constituents of summer savory and thyme white plant essential oils had weak or no antifungal activities at 2.5 and 1.25 mg/paper disc concentration. In a test with *R. solani*, thymol and carvacrol showed a 100% inhibition rate at concentration more than 0.625 mg/paper disc concentration. Inhibition was 78.64% for thymol and 81.96% for carvacrol at 0.3125 mg/paper disc concentration and 51.28% and 55.50% at 0.15625 mg/paper disc concentration, respectively. Fumigant antifungal activities of terpinen-4-ol, (+)-α-pinene, (−)-α-pinene, and linalool ranged from 71.30–42.62% at 2.5 mg/paper disc concentration, and less than 40% at 1.25 mg/paper disc concentration. Other constituents of summer savory and thyme white essential oils showed weak or no fumigant antifungal activities against *R. solani* at 2.5 mg/paper disc concentration. In our study, thymol and carvacrol showed very strong fumigant antifungal activities against the two phytopathogenic fungi. Thymol and its isomer carvacrol are phenolic monoterpenoids found in the Lamiaceae family. Reported biological effects for thymol and carvacrol include antispasmodic, antioxidant, anti-inflammatory, and anticarcinogenesis activity [21,22]. Among identified constituents of thyme white and summer savory plant essential oils, the antifungal activity of phenolic monoterpenes (thymol and carvacrol) was much higher than the activity of monoterpene hydrocarbons (α-pinene, camphene, β-pinene, myrcene, α-terpinene, *p*-cymene, limonene, *γ*-terpien), alcohols (terpenin-4-ol and linalool) and sesquiterpenes (*β*-caryophyllene, caryophyllene oxide). These results were very similar to the results of Kim et al. [15], that phenol (thymol, carvacrol, phenylpropanoids) compounds generally exhibited strong fumigant antifungal activity against the two phytopathogenic fungi, *Phytophthora cactorum,* and *Cryponectria parasitica*, compared to hydrocarbon monoterpenes.

Carvacrol and thymol have delocalized electron and hydroxyl groups. Utlee et al. [23] suggested that the hydroxyl group and delocalized electron of carvacrol and thymol are essential for antifungal activity. Delocalized electrons in double bonds facilitate carvacrol and thymol releasing cations from hydroxyl groups when undissociated carvacrol and thymol diffuse through the cytoplasmic membrane to the cytoplasm. Carvacrol and thymol could then return to the outside of cells undissociated by carrying potassium or other cation from the cytoplasm. This hypothesis is supported by the observation of the efflux of K^+^ and the influx of H^+^ in *Bacillus cereus* treated with carvacrol [24]. The decrease in the ΔpH might eliminate the proton motive force for adenosine triphosphate (ATP) synthesis in the cell. Utlee et al. [23] reported that the antifungal activities of carvacrol methyl ester and *p*-cymene, a precursor of carvacrol, were lower than carvacrol because of a lack of hydroxyl groups. Antifungal activity was also lower for menthol than carvacrol because of a lack of delocalized electrons in menthol. Our study had the same result. Phenolic monoterpenes with hydroxyl groups and delocalized electrons exhibited very strong fumigant antifungal activity against *R. querqus-mongolicae* and *R. solani* compared to compounds without hydroxyl groups and delocalized electrons. The only difference in the chemical structure of thymol and carvacrol is the position of hydroxyl groups. In our study, thymol had higher antifungal activity than carvacrol against *R. querqus-mongolicae*. However, no significant difference was observed in the antifungal activity against *R. solani*. Lambert et al. [25] reported the hydroxyl group position of thymol and carvacrol did not cause differences in antimicrobial activity. However, positional differences in the hydroxyl group of thymol and carvacrol caused slight changes in the antifungal activity against *Candida* isolates in another study [26]. Our study and previous studies indicate that the effect on antifungal activity on the hydroxyl position of thymol and carvacrol varies according to fungal species.

### 3.4. Effect of Carvacrol and Thymol on Reactive Oxygen Species Generation

**Figure 2:**
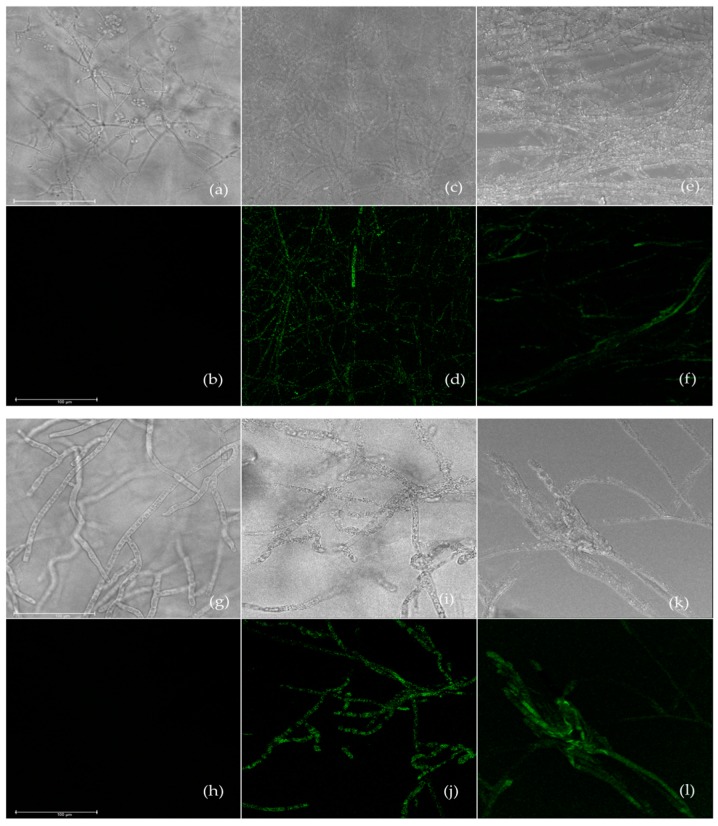
Confocal laser scanning microscopy images of ROS generation. Bright mode (**a**) and confocal image (**b**) of untreated *R. quercus-mongolicae*. Bright mode (**c**) and confocal image (**d**) of *R. quercus-mongolicae* treated with thymol. Bright mode (e) and confocal image (f) of *R. quercus-mongolicae* treated with carvacrol. Bright mode (**g**) and confocal image (h) of untreated *R. solani*. Bright mode (**i**) and confocal image (**j**) of *R. solani* treated thymol. Bright mode (k) and confocal image (**l**) of *R. solani* treated with carvacrol. Scale bar = 50 μm.

Intracellular ROS generation of *R. querqus-mongolicae* and *R. solani* treated with carvacrol and thymol is in Figure 2. A strong fluorescence signal was detected from *R. querqus-mongolicae* and *R. solani* treated with carvacrol and thymol (Figure 2d,f,j,l) compared to controls (Figure 2b,h). Representative molecules of ROS include hydroxyl radical (^•^OH), superoxide anion (O_2_^−^), and hydrogen peroxide (H_2_O_2_) [27]. ROS are important in cell signaling and homeostasis. However, the antibacterial or antifungal action of many antibiotics and antifungal agents is related to the accumulation of ROS in bacteria and fungi, especially in *Candida* species [27,28,29,30]. Studies suggest that antifungal agents induce ROS formation in filamentous fungi such as *Rhizoctonia*, *Fusarium*, and *Aspergillus* species [31,32]. ROS-related antifungal activities of plant essential oils or their constituents are documented [33,34]. Shen et al. [33] reported that thymol treatment induces ROS and nitric oxide in *Aspergillus flavus* spores, causing spore death. Tian et al. [34] demonstrated that ROS is an important mediator of the antifungal action of dill oil against *A. flavus*. Our study and prior studies indicated that thymol and carvacrol induce ROS in *R. querqus-mongolicae* and *R. solani*, and accumulation of ROS might cause fungal cell damage. However, further research is necessary to elucidate the pathway of ROS generation by thymol and carvacrol in *R. querqus-mongolicae* and *R. solani*.

### 3.5. Effect of Carvacrol and Thymol on Cell Membrane Integrity

**Figure 3:**
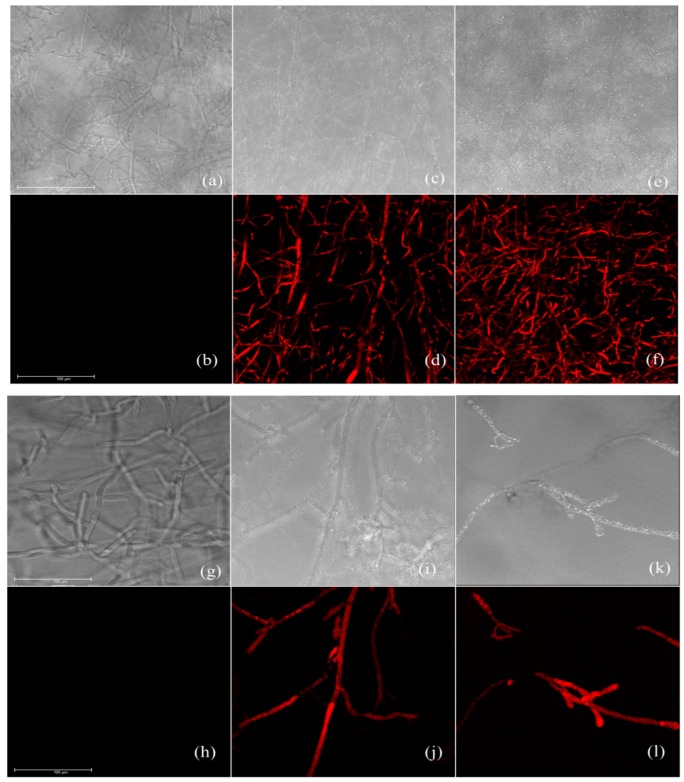
Confocal laser scanning microscopy images of cell membrane integrity. Bright mode (**a**) and PI stained (**b**) of untreated *R. quercus-mongolicae*. Bright mode (**c**) and PI-stained (**d**) of *R. quercus-mongolicae* treated with thymol. Bright mode (**e**) and PI stained (**f**) of *R. quercus-mongolicae* treated with carvacrol. Bright mode (**g**) and PI-stained (**h**) of untreated *R. solani*. Bright mode (**i**) and PI-stained (**j**) of *R. solani* treated with thymol. Bright mode (**k**) and PI-stained (**l**) of *R. solani* treated with carvacrol. Scale bar = 50 μm.

CLSM images of *R. querqus-mongolicae* and *R. solani* stained with PI are shown in Figure 3. A strong fluorescence signal indicating dead cellc stained with PI was seen for *R. querqus-mongolicae* and *R. solani* treated with carvacrol and thymol (Figure 3d,f,j,l). No similar fluorescence signal was observed in untreated *R. querqus-mongolicae* and *R. solani* (Figure 3b,h). PI molecules penetrate only corrupted cell membranes to stains cells [35]. This dye is used for testing plasma membrane integrity [36]. Xu et al. [37] observed PI-stained mycelia of *Alternaria aternata* treated with cinnamaldehyde, but no staining in untreated *A. aternata*. CLSM images of *R. querqus-mongolicae* and *R. solani* stained with PI confirmed that thymol and carvacrol damaged the cell membranes of *R. querqus-mongolicae* and *R. solani.* Based on our results, we concluded that ROS generated by thymol and carvacrol damaged cell membranes of *R. querqus-mongolicae* and *R. solani*, and this might cause cell death. However, we did not determine the ROS generation pathway in this study. Future studies on ROS generation pathways are necessary to determine the antifungal mode of action of thymol and carvacrol against *R. querqus-mongolicae* and *R. solani*.

## 4. Conclusions

Thyme white and summer savory plant essential oils showed strong fumigant antifungal activity against two phytopathogenic fungi, *R. quercus-mongolicae* and *R. solani*. Among the identified constituents from the two plant essential oils, fumigant antifungal activities of two phenolic monoterpenes, thymol and carvacrol, were stronger than the activities of monoterpene hydrocarbons, alcohols, and sesquiterpenes. We concluded that intracellular ROS generated by thymol and carvacrol damaged cell membranes and this might have caused cell death of *R. quercus-mongolicae* and *R. solani.* The practical use of plant essential oils and their constituents as control agents against the phytopathogenic fungi *R. quercus-mongolicae* and *R. solani* will require the development of formulations and toxicity tests for non-target organisms.

## Figures and Tables

**Table 1:** List of Lamiaceae plant essential oils.

Common Name	Scientific Name	Source	Part
Basil	*Ocimum basilicum* L.	Jinarome	Leaves
Hyssop	*Hyssopus officinalis* L.	Osahdi	plants
Lavender	*Lavandula angustifolia* Mill.	Jinarome	Flowers
Marjoram	*Origanum majorana* L.	Jinarome	Fruits
Patchouli	*Pogostemon cablin* Benth.	Oshadi	Leaves
Pennyroyal	*Mentha pulegium* L.	Jinarome	Plants
Spanish Sage	*Salvia lavandulaefolia* Vahl.	Jinarome	Flowers
Spearmint	*Mentha spicata* L.	Jinarome	Flowering plants
Summer Savory	*Satureja hortensis* L.	Osahdi	Blossom/plants
Thyme White	*Thymus vulgaris* L.	Jinarome	Leaves

**Table 2:** Fumigant antifungal activities of Lamiaceae plant essential oils against *R. quercus-mongolicae*.

Essential Oils.	Inhibition Rate (%, mean ± SE)					
	10 ^1^	5	2.5	1.25	0.625	0.3125
Basil	58.42 ± 1.21b ^2^	0d	- ^3^	-	-	-
Hyssop	27.72 ± 2.38c	0d	-	-	-	-
Lavender	37.46 ± 8.53c	0d	-	-	-	-
Marjoram	37.30 ± 5.77c	0d	-	-	-	-
Patchouli	24.60 ± 4.27c	0d	-	-	-	-
Pennyroyal	72.38 ± 2.29b	44.40 ± 0.35c	34.62 ± 2.82b	27.96 ± 2.70c	0b	-
Spanish sage	59.06 ± 1.77b	0d	-	-	-	-
Spearmint	91.76 ± 0.75a	57.50 ± 1.55b	37.06 ± 1.94b	15.98 ± 2.29d	0b	-
Summer savory	100a	100a	100a	91.50 ± 1.60a	54.40 ± 0.78a	34.86 ± 4.01
Thyme white	100a	100a	100a	73.52 ± 0.64b	50.18 ± 2.79a	29.30 ± 2.01
	F_9,40_ = 60.81	F_9,40_ = 7110	F_3,16_ = 469	F_3,16_ = 60.81	F_3,16_ = 60.81	F_1,8_ = 60.81
	*p* < 0.0001	*p* < 0.0001	*p* < 0.0001	*p* < 0.0001	*p* < 0.0001	*p* = 0.25

^1^ mg/paper disc. ^2^ Means within a column followed by the same letters are not significantly different (Tukey’s HSD test). ^3^ Not tested.

**Table 3:** Fumigant antifungal activities of Lamiaceae plant essential oils against *R. solani*.

Essential Oils	Inhibition Rate (%, mean ± SE)					
	10 ^1^	5	2.5	1.25	0.625	0.3125
Basil	100a ^2^	54.40 ± 1.04e	20.20 ± 1.24d	0d	-	-
Hyssop	63.96 ± 1.33d	36.64 ± 1.54e	11.96 ± 1.14e	0d	-	-
Lavender	86.38 ± 0.64b	62.86 ± 1.13c	19.28 ± 1.98d	0d	-	-
Marjoram	0e	- ^3^	-	-	-	-
Patchouli	0e	-	-	-	-	-
Pennyroyal	100a	87.96 ± 0.90b	36.42 ± 2.29c	21.96 ± 0.96c	17.70 ± 0.70c	0c
Spanish sage	73.08 ± 0.95c	57.50 ± 0.96d	23.52 ± 0.88d	0d	-	-
Spearmint	100a	91.30 ± 0.89b	44.18 ± 1.12b	25.28 ± 1.07c	0d	-
Summer savory	100a	100a	100a	91.76 ± 2.07a	82.18 ± 1.00a	41.98 ± 0.81b
Thyme white	100a	100a	100a	85.72 ± 1.17b	63.30 ± 1.25b	54.38 ± 2.53a
	F_9,40_ = 5264	F_7,32_ = 631.3	F_7,32_ = 730.9	F_7,32_ = 1562	F_3,16_ = 1926	F_2,12_ = 346.4
	*p* < 0.0001	*p* < 0.0001	*p* < 0.0001	*p* < 0.0001	*p* < 0.0001	*p* < 0.001

^1^ mg/paper disc. ^2^ Means within a column followed by the same letters are not significantly different (Tukey’s HSD test). ^3^ Not tested.

**Table 4:** Chemical composition of summer savory and thyme white plant essential oils.

No.	Compound	Retention Index	Composition Rate (%)	
		DB-5	Summer Savory	Thyme White
1	α-Pinene	930	1.34	5.25
2	Camphene	946	- ^1^	1.41
3	*β*-Pinene	973	0.43	-
4	Myrcene	989	1.81	3.35
5	*α*-Terpinene	1014	3.51	-
6	*p*-Cymene	1022	10.72	30.16
7	Limonene	1027	0.63	1.03
8	*γ*-Terpinene	1057	34.63	11.44
9	Linalool	1100	-	9.49
10	Terpinen-4-ol	1179	0.50	-
11	Thymol	1290	0.21	25.32
12	Carvacrol	1298	39.84	3.46
13	*β*-Caryophyllene	1415	2.40	1.75
14	Caryophyllene oxide	1577	-	1.15
Sum			96.04	93.81

^1^ Not detected.

**Table 5:** Fumigant antifungal activities of constituents from summer savory and thyme white essential oils against *R. quercus-mongolicae*.

Compounds	Inhibition Rate (%, mean ± SE)				
	2.5 ^1^	1.25	0.625	0.3125	0.15625
(+)-α-Pinene	0e ^2^	- ^3^	-	-	-
(−)-α-Pinene	0e	-	-	-	-
(+)-Camphene	0e	-	-	-	-
(−)-Camphene	0e	-	-	-	-
(+)-β-Pinene	0e	-	-	-	-
(−)-β-Pinene	0e	-	-	-	-
Myrcene	0e	-	-	-	-
α-Terpinene	0e	-	-	-	-
p-Cymene	0e	-	-	-	-
(+)-Limonene	0e	-	-	-	-
(−)-Limonene	0e	-	-	-	-
γ-Terpinene	0e	-	-	-	-
Linalool	15.48 ± 2.25d	0d	-	-	-
Terpinen-4-ol	74.84 ± 0.89b	59.28 ± 3.37b	47.06 ± 1.47c	21.74 ± 1.04c	0b
Thymol	100a	100a	100a	53.30 ± 0.92a	28.40 ± 2.45a
Carvacrol	100a	100a	81.28 ± 2.20b	30.68 ± 2.09b	0b
β-Caryophyllene	0e	-	-	-	-
Caryophyllene oxide	46.18 ± 2.91c	20.64 ± 2.56c	10.38 ± 1.67d	0d	-
	F_17,72_ = 1601	F_4,20_ = 578.2	F_3,16_ = 534.8	F_3,16_ = 310.1	F_2,12_ = 134.7
	*p* < 0.0001	*p* < 0.0001	*p* < 0.0001	*p* < 0.0001	*p* < 0.0001

^1^ mg/paper disc. ^2^ Means within a column followed by the same letters are not significantly different (Tukey’s HSD test). ^3^ Not tested.

**Table 6:** Fumigant antifungal activities of constituents from summer savory and thyme white essential oils against *R. solani*.

Compounds	Inhibition Rate (%, mean ± SE)				
	2.5 ^1^	1.25	0.625	0.3125	0.15625
(+)-α-Pinene	47.52 ± 1.84c ^2^	33.52 ± 1.37b	21.52 ± 1.35b	0b	-
(−)-α-Pinene	47.30 ± 1.88c	18.38 ± 1.10c	0d	-	-
(+)-Camphene	0f	- ^3^	-	-	-
(−)-Camphene	0f	-	-	-	-
(+)-β-Pinene	0f	-	-	-	-
(−)-β-Pinene	0f	-	-	-	-
Myrcene	0f	-	-	-	-
α-Terpinene	0f	-	-	-	-
p-Cymene	0f	-	-	-	-
(+)-Limonene	22.64 ± 0.44d	0d	-	-	-
(−)-Limonene	0f	-	-	-	-
γ-Terpinene	0f	-	-	-	-
Linalool	42.62 ± 1.74c	0d	-	-	-
Terpinen-4-ol	71.30 ± 1.84b	32.40 ± 1.29b	15.28 ± 1.32c	0b	-
Thymol	100a	100a	100a	78.64 ± 1.34a	51.28 ± 6.98
Carvacrol	100a	100a	100a	81.96 ± 3.23a	55.50 ± 0.92
β-Caryophyllene	15.74 ± 1.77e	0d	-	-	-
Caryophyllene oxide	0f	-	-	-	-
	F_17,72_ = 1338	F_7,32_ = 2997	F_4,20_ = 3331	F_3,16_ = 703.9	F_1,8_ = 0.359
	*p* < 0.0001	*p* < 0.0001	*p* < 0.0001	*p* < 0.0001	*p* = 0.566

^1^ mg/paper disc. ^2^ Means within a column followed by the same letters are not significantly different (Tukey’s HSD test). ^3^ Not tested.
